# Agreement Between Methods Assessing Changes in Plasma Volume During Fluid Therapy—A Post Hoc Analysis of a Randomized Trial

**DOI:** 10.1111/aas.70271

**Published:** 2026-06-04

**Authors:** David Grubb, Svajunas Statkevicius, Johan Bonnevier, Björn Bark, Peter Bentzer

**Affiliations:** ^1^ Department of Cardiothoracic Anesthesia and Intensive Care Skåne University Hospital Lund Sweden; ^2^ Department of Clinical Sciences Lund, Anesthesiology and Intensive Care Lund University Lund Sweden; ^3^ Department of Intensive and Perioperative Care Skåne University Hospital Lund Sweden; ^4^ Department of Intensive and Perioperative Care Skåne University Hospital Malmö Sweden; ^5^ Department of Clinical Sciences Malmö, Anesthesiology and Intensive Care Lund University Malmö Sweden

**Keywords:** fluid therapy, hematocrit, plasma volume

## Abstract

Volume status of a patient is difficult to assess clinically. Methods to measure plasma volume as well as changes in plasma volume in connection with fluid therapy are therefore valuable for research purposes and could potentially be used bedside to guide fluid therapy in individual patients. The objective of the present study was to evaluate hematocrit as a marker of changes in plasma volume and an anthropometric formula to estimate absolute plasma volume against the reference method, plasma volume measurements using radiolabeled albumin. A total of 64 postoperative patients received 10 mL/kg of albumin. The plasma volumes were measured with the reference method (radiolabeled albumin) at baseline, after 30 and 180 min. Plasma volumes were compared with plasma volumes derived from either the baseline measurements and subsequent changes in hematocrit (calibrated method) or from an anthropometric formula and subsequent hematocrit changes (anthropometric method). Bland–Altman plots were used to test agreements between methods. The mean difference in plasma volume between the anthropometric and the reference method at baseline was −0.1 mL/kg (95% CI –2.1 to 1.8) with lower and upper LOA of −18.0 and 17.0 mL/kg. The agreement remained essentially unchanged after fluid therapy (mean difference −0.1 mL/kg (95% CI –1.8 to 1.5) with LOAs of −18.0 and 17.0 mL/kg). The mean difference between the calibrated and reference methods was −0.1 mL/kg (95% CI –0.9 to 0.7) with LOAs of −9.4 and 9.2 mL/kg. The anthropometric formula yields low precision in predicting plasma volumes in postsurgical patients. Changes in hematocrit during fluid therapy cannot replace the reference method to assess changes in plasma volume in the individual patient because of the imprecision.

**Editorial Comment:**

Clinical appreciation of plasma volume is relevant for assessing treatment where intravenous fluid resuscitation is involved. This analysis, using a reference method for plasma volume assessment, and comparing to simpler methods to estimate the same, demonstrates that there are important limitations with some simpler and readily acceptable methods to perform this estimation.

## 1 Introduction

The main objective of fluid therapy is to increase preload in order to ensure adequate oxygen delivery to the tissues. However, overly aggressive fluid administration may result in tissue oedema, which may impede oxygen delivery and adversely affect outcome [1, 2, 3]. It is well known that the volume status of a patient is difficult to assess based on clinical signs [4]. Methods to measure the effect of fluid therapy on circulating plasma volume are therefore valuable for research purposes. Potentially, measurements of changes in plasma volume during fluid therapy could also be used bedside to complement existing dynamic parameters to guide fluid therapy in individual patients.

The gold standard for measurement of plasma volume is considered to be measurement of the initial distribution volume of radiolabeled albumin [5, 6]. However, decreased demand for radiochemicals has increased production costs, making multiple determinations of plasma volume in the same subject very costly. Moreover, serial measurements of plasma volume using radiochemicals are labor intensive and logistically challenging. A methodology that reduces costs and labor is therefore warranted.

Using changes in hematocrit as a measure of changes in plasma volume is cheaper than radiolabeled albumin, eliminates radiation exposure and can be performed using widely available analyzers with a high precision [7]. However, large vessel hematocrit differs from that in small vessels [8, 9]. Thus, if the ratio of small to large vessel hematocrit changes during fluid therapy, induction of anesthesia or other therapeutic interventions, changes in hematocrit may not accurately reflect changes in plasma volume. Also, if bleeding occurs during the experimental period, changes in hematocrit will not be an accurate measure of plasma volume change. In addition, hematocrit measurements can only be used to calculate relative changes in plasma volume, not to determine the absolute plasma volume.

Based on these considerations the primary objective of the present study was to investigate whether hematocrit measurements following one baseline measurement of plasma volume using ^125^I‐labeled albumin can be used to calculate changes in plasma volume during and after intravenous fluid therapy in postoperative patients. The secondary objective was to assess whether hematocrit measurements following calculation of baseline plasma volume using an anthropometric formula can be used to calculate changes in plasma volume during and after intravenous fluid therapy in postoperative patients. Plasma volumes measured using ^125^I‐labeled albumin were used as the reference method. The agreements between the methods were evaluated using Bland–Altman plots.

## 2 Methods

### 2.1 Participants

Data included in the present study were collected from the randomized controlled trial (RCT), “The importance of albumin infusion rate for plasma volume expansion following major abdominal surgery” (AIR) cohort of participants. The AIR trial was approved by the regional ethical vetting board in Lund, Sweden (Dnr 2014/15) and by the Medical Product Agency in Sweden (EudraCT nr: 2013–004446‐42). A detailed experimental protocol and the main findings of the trial have been presented elsewhere [10, 11]. Patients who had undergone a non‐emergent Whipple's operation or major gynecological cancer surgery that were ≥ 40 years old, had given written consent and showed signs of hypovolemia within 5 h after arrival to the post operative care unit (PACU) were eligible for inclusion. Participants were randomized to receive 5% albumin (CSL Behring) at a dose of 10 mL/kg predicted body weight [12] in either 30 (fast group) or 180 min (slow group). Plasma volumes were measured as described below immediately before administration of albumin (baseline) and at 30 and 180 min after the start of the albumin infusion. Three different investigators were involved bedside in the project and injections and blood sampling in each patient was performed by one of these investigators.

### 2.2 Measurement of Plasma Volume Using Multiple Injections of ^125^I‐Human Serum Albumin (Reference Method)

Plasma volumes were determined by measuring the distribution volume of ^125^I‐human serum albumin (HSA) (SERALB, CIS bio international, France). The distribution volume was calculated as the injected dose divided by the change in plasma activity. The injected doses ranged between 0.19 and 0.23 MBq. The exact amount of tracer that was injected was calculated by subtracting the amount of tracer remaining in the respective syringe after injection from the amount of tracer in the syringe prior to injection. ^125^I‐HSA was injected in a peripherally placed intravenous 18G cannula 10 min before the samples for determination of plasma activity were drawn. Blood samples for determination of plasma activity were drawn 5 min before the second and third injection of ^125^I‐HSA to allow for calculation of change in plasma activity. Blood samples for analysis of plasma activity of ^125^I‐HSA were collected from an arterial line placed in the radial artery in a 5 mL EDTA containing vial (BD Vacutainer, Becton, Stockholm, Sweden). Samples were centrifuged and transferred into pre‐weighed vials. The volume of plasma in each vial was then determined by measuring change in weight by a high precision scale (Mettler‐Toledo AE 200, Stockholm, Sweden). The activity of ^125^I‐HSA in the vials was measured in a gamma counter (PerkinElmer 1480 Wizard; PerkinElmer, Waltham, MA, USA). Plasma volumes were normalized to the predicted body weight of the participants [12].

### 2.3 Measurement of Plasma Volume Using One Injection of ^125^I‐Human Serum Albumin Followed by Measurements of Hematocrit (Calibrated Method)

Plasma volume at baseline was determined as described above. Blood samples were collected in heparinized syringes from the radial artery line immediately prior to the start of the albumin infusion and 5 min before the second and third injection of ^125^I‐HSA. Hematocrit was then determined in a standard blood gas analyzer (Radiometer 800, Copenhagen, Denmark) according to the instructions of the manufacturer. After hemolysation the concentration of hemoglobin (ctHb) is determined spectrophotometrically. The hematocrit is then derived from the ctHb via an internal algorithm. The coefficient of variation for this analysis is ≤ 1.2% [13].

Intravascular mass of hemoglobin, red cell volume and the ratio of arterial to whole body hematocrit were assumed to remain constant throughout the experiment. Based on these assumptions, a change in hematocrit reflects a change in plasma volume. The plasma volume (PV) at time (t) is then given by the equation [14]:

$$\mathrm{PV}\;\left(t\right)={\mathrm{PV}}_{\mathrm{bl}}\times \left(1+\frac{\left(\frac{{\mathrm{Hct}}_{\mathrm{bl}}}{{\mathrm{Hct}}_{t}}-1\right)}{\left(1-{\mathrm{Hct}}_{\mathrm{bl}}\right)}\right)$$

where PV_bl_ represents plasma volume at baseline, Hct_bl_ represents hematocrit at baseline and Hct_t_ represents hematocrit at either 30 or 180 min after the start of fluid therapy.

### 2.4 Prediction of Plasma Volume at Baseline Using an Anthropometric Formula Followed by Measurements of Hematocrit (Anthropometric Method)

Baseline blood volume was predicted by the Nadler formula [15]:

$${\mathrm{BV}}_{\mathrm{bl}}=a\times h^{3}+b\times w+c$$

where BV_bl_ is predicted blood volume at baseline, *h* is height (m), *w* is actual preoperative weight (kg), and *a*, *b*, and *c* represent sex‐specific constants. The predicted plasma volume (PPV) was obtained by:

$$\mathrm{PPV}={\mathrm{BV}}_{\mathrm{bl}}\times \left(1-\mathrm{Hct}\right)$$

where Hct is baseline hematocrit. The predicted plasma volumes at 30 and 180 min were calculated using hematocrit measurements as described for the calibrated method.

### 2.5 Statistics

All analyses were performed without knowledge of the treatment allocation of respective patients. Only patients with complete data sets were included in the analysis. Accuracy of serial changes in hematocrit to estimate changes in plasma volume was evaluated as suggested by Bland and Altman for measurements with more than one observation per patient [16]. The assumption that the variance of the repeated measurements within each subject is independent of the mean of each subject was assessed graphically by plotting the standard deviation of the measurement differences against the mean of the two methods. A difference‐in‐difference analysis was employed together with a Bonferroni correction to investigate whether the infusion rate impacted the agreement between the reference, the calibrated and the anthropometric methods. *p* values < 0.05 were considered to be significant. All tests were two‐tailed. Data are presented as mean and standard deviation or median and interquartile range as appropriate. All analyses were performed in Stata 18 and R v.4.4.3.

## 3 Results

### 3.1 Patient Characteristics

A total of 64 patients, 31 in the fast and 33 in the slow infusion group, furnished 128 pairs of measurements in the calibrated vs. reference and in the anthropometric vs. reference group. These are the same participants included in the main report of the AIR trial [11]. Patient characteristics are presented in Table 1. The plot of standard deviation of differences against the mean plasma volume within each patient did not suggest that the differences were dependent on the mean plasma volumes of patients (Figures S1a and S1b).

**TABLE 1:** Demographics and baseline data of the cohort.

Female	41 (64)
Age, years	69 (64–73)
Actual weight, kg	70.9 (62.7–85)
Predicted body weight, kg	59.2 (55.6–70.9)
Noradrenaline infusion	4 (6)
Epidural analgesia	59 (92)
Type of surgery	
Whipple	35 (55)
Gynecological surgery	29 (45)
Baseline hemodynamics and laboratory data	
HR, beats per minute	85 (74–94)
Systolic blood pressure, mm Hg	113 (99–126)
MAP, mm Hg	77 (68–87)
CVP, mm Hg	3 (0–7)
Hb, g/L	115 (106–124)
Lactate, mmol/L	2.4 (1.6–3.1)

*Note:* Baseline refers to data immediately prior to measurement of plasma volumes. Data are presented as number (%) or median (IQR) as appropriate.

Abbreviations: CVP, central venous pressure, Hb, hemoglobin, HR, heart rate, IQR, interquartile range; MAP, mean arterial pressure.

### 3.2 Plasma Volume Measurements

**FIGURE 1:**
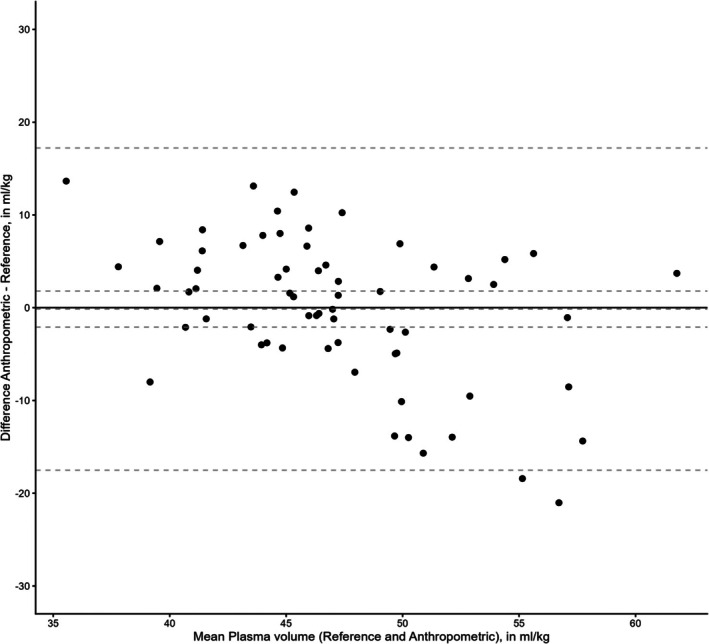
Bland–Altman plot of the anthropometric and reference methods at baseline. Hatched lines are mean difference with 95% CI and upper and lower limits of agreement. Plasma volumes are normalized to predicted body weights. *N* = 64.

The reference plasma volume at baseline was 47.3 ± 7.9 mL/kg (Table 2). After the start of fluid therapy, the reference PV increased to 53.0 ± 8.4 at 30 min and to 54.3 ± 7.9 mL/kg at 180 min (Table 2). Using the calibrated method, the PV increased to 51.2 ± 9.0 at 30 min and to 55.9 ± 8.9 mL/kg at 180 min according to changes in hematocrit (Table 2). Using the anthropometric method, the plasma volume at baseline was 47.1 ± 5.0 mL/kg, which increased to 51.1 ± 6.9 at 30 min and to 55.9 ± 7.0 at 180 min according to changes in hematocrit (Table 3). The mean difference in plasma volume between the anthropometric and the reference method at baseline was −0.1 mL/kg (95% CI −2.1 to 1.8 mL/kg) with lower and upper LOA of −18.0 and 17.0 mL/kg (Figure 1).

**TABLE 2:** Plasma volumes for the reference and calibrated methods.

Group	*N*	PV bl_ref_	PV 30_ref_	PV 30_cal_	PV 180_ref_	PV 180_cal_
		(mL/kg)	(mL/kg)	(mL/kg)	(mL/kg)	(mL/kg)
30 (fast)	31	47.7 ± 6.3	55.8 ± 5.9	55.3 ± 6.4	54.2 ± 6.7	56.5 ± 7.6
180 (slow)	33	46.9 ± 9.2	50.3 ± 9.5	47.4 ± 9.4	54.3 ± 8.9	55.2 ± 10.0
All	64	47.3 ± 7.9	53.0 ± 8.4	51.2 ± 9.0	54.3 ± 7.9	55.9 ± 8.9

*Note:* ref = reference method; cal = calibrated method. Data are mean ± SD.

Abbreviations: bl, baseline; PV, plasma volume.

**TABLE 3:** Plasma volumes for the anthropometric method.

Group	*N*	PV bl_anthr_	PV 30_anthr_	PV 180_anthr_
		(mL/kg)	(mL/kg)	(mL/kg)
30 (fast)	31	47.2 ± 4.9	54.8 ± 6.2	56.1 ± 7.3
180 (slow)	33	47.1 ± 5.2	47.6 ± 5.7	55.7 ± 6.8
All	64	47.1 ± 5.0	51.1 ± 6.9	55.9 ± 7.0

*Note:* anthr = anthropometric method; Data are mean ± SD.

Abbreviations: bl, baseline; PV, plasma volume.

**FIGURE 2:**
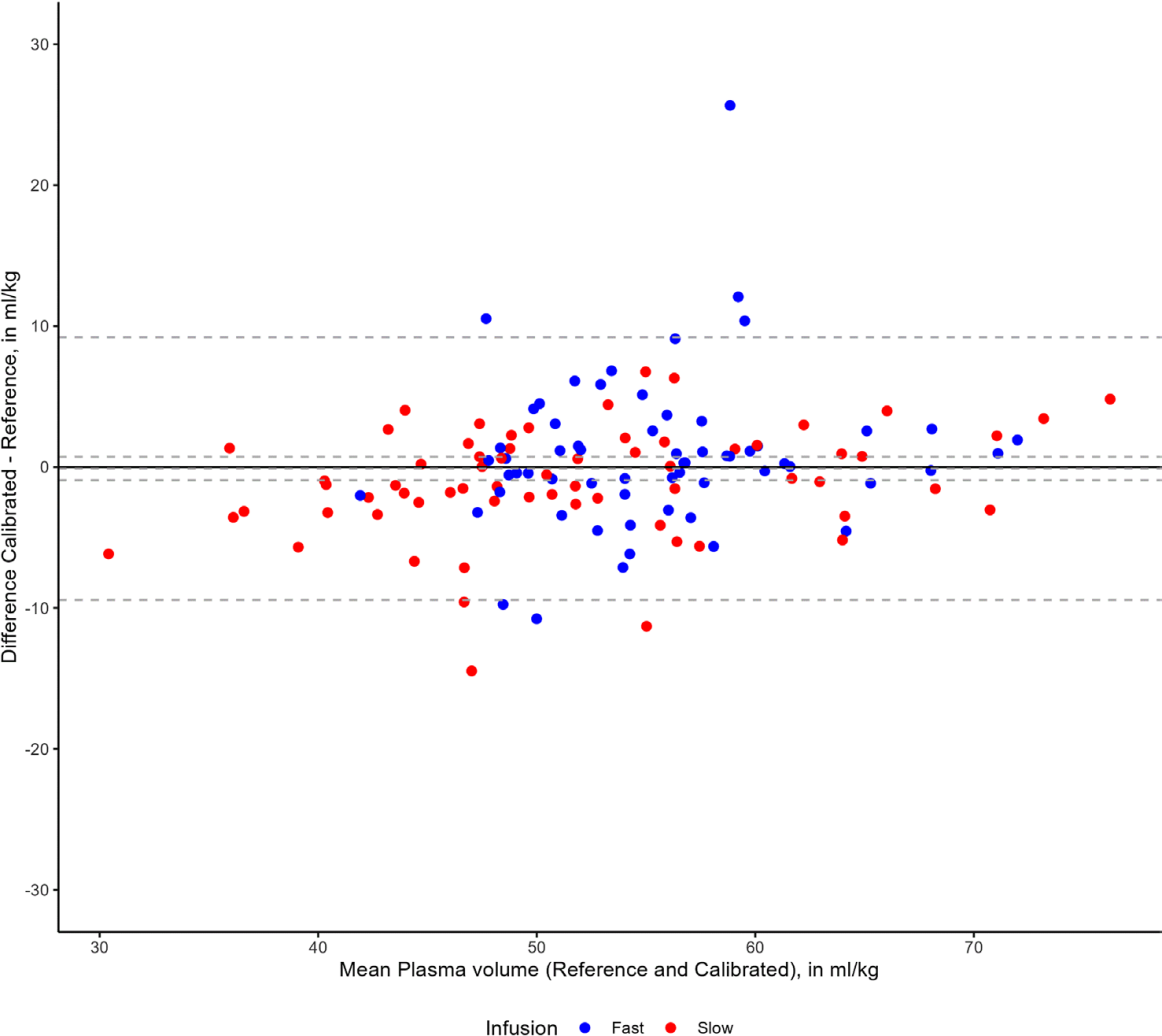
Bland–Altman plot of the calibrated and reference methods. Hatched lines are mean difference with 95% CI and upper and lower limits of agreement. Plasma volumes are normalized to predicted body weights. *N* = 128.

**FIGURE 3:**
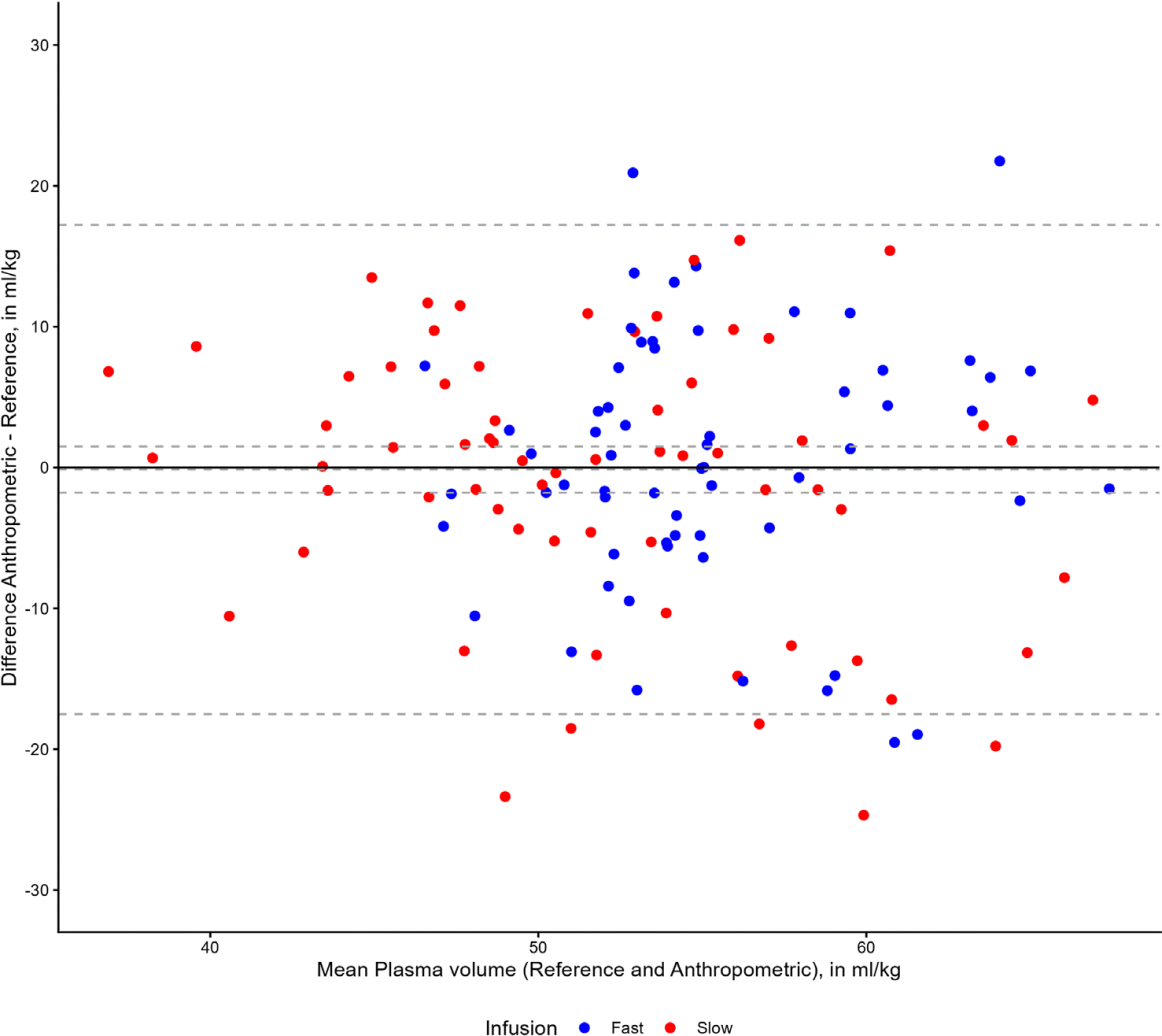
Bland–Altman plot of the anthropometric and reference methods. Hatched lines are mean difference with 95% CI and upper and lower limits of agreement. Plasma volumes are normalized to predicted body weights. *N* = 128.

The pooled mean difference between the plasma volumes using the calibrated and reference methods at 30 and 180 min was −0.1 mL/kg (95% CI −0.9 to 0.7 mL/kg) with lower and upper LOA of −9.4 and 9.2 mL/kg (Figure 2). The pooled mean difference between the plasma volumes using the anthropometric and reference methods at 30 and 180 min was −0.1 mL/kg (95% CI −1.8 to 1.5 mL/kg) with lower and upper LOA of −18.0 and 17.0 mL/kg (Figure 3).

The difference‐in‐difference analysis of the calibrated vs. the reference method showed an overall difference in agreement between the slow and fast infusion groups of −1.9 mL/kg (95% CI −3.5 to −0.3 mL/kg; *p* = 0.019) (Table S1). The effect of the infusion rate, however, was not significant neither at 30 nor at 180 min following correction for multiple comparisons (Table S1). There was no difference in agreement between the slow and fast infusion groups in the anthropometric vs. the reference methods (Table S1).

## 4 Discussion

The main findings of the present study were that the mean differences between plasma volumes during and after fluid therapy determined by the reference method compared with the calibrated and anthropometric methods were very small. In contrast, the limits of agreement between the methods were wide, especially between the reference and the anthropometric method.

The limits of agreement are dependent on the precision of both the reference method and the method that is being compared. The precision of the reference method was not determined in the present study, but in previous studies the coefficient of variance was 2%–5% [4, 5]. Assuming a similar precision here, the wide limits are more likely caused by the anthropometric and calibrated methods. Several explanations are possible. As earlier mentioned, both the calibrated and the anthropometric methods are dependent on a constant ratio between whole body hematocrit and large vessel hematocrit—an assumption that may not be valid during fluid therapy [17]. Given that the participants in the trial were postoperative after major surgery, another potential source of error of using hematocrit is bleeding. Although no bleeding was diagnosed, smaller blood losses may have gone undetected and could therefore also have contributed to randomly appearing differences between the methods. Admittedly, since it is difficult to ascertain the absence of bleeding in most postoperative settings, the use of hematocrit here is inherently sensitive to this source of error.

The wider limits of agreement of the anthropometric method compared to the calibrated method are likely explained by the wide limits of agreement already at baseline. There are a number of possible explanations for why the anthropometric method performed poorly in predicting the baseline plasma volume. First, there might be a difference in body composition between the study and the Nadler populations. This possibility is suggested by the difference of approximately 12 kg between the actual and predicted weight of the participants in the present study. Furthermore, the anthropometric method is based on equations derived from plasma volumes measured using radiolabeled albumin under stable conditions [15] which have not been validated in a post‐surgery setting. Several studies have suggested that changes in vascular tone will decrease capillary hydrostatic pressure which in turn will transiently favor transcapillary absorption and an increase in plasma volume [7, 18, 19]. Given that a majority of participants in the AIR trial were treated with epidural analgesia, which will decrease vascular tone and potentially alter capillary hydrostatic pressure [20], it is possible that the plasma volumes at baseline are altered as a physiological response to epidural analgesia. Also, major surgery will induce a systemic inflammatory response syndrome (SIRS) which may influence vascular tone and consequently capillary hydrostatic pressure to a variable degree. Lastly, peroperative bleeding, other fluid losses as well as fluid therapy will alter plasma volume at baseline.

Following determination of the agreement between the reference method and the calibrated and anthropometric methods a relevant question is whether the latter methods can replace the reference method. As stated by Bland and Altman [21] the answer to this question is dependent on the context in which we would like to use the methods. In this particular clinical setting, the results suggest that, although the mean differences were small, the wide limits of agreements between methods preclude the replacement of the reference method with hematocrit as a measure of plasma volume change. As shown, the LOAs between the reference and calibrated methods were similar to or higher than the fluid dose administered (10 mL/kg). This suggests that any conclusion regarding the clinical effect of the fluid therapy in the individual patient is uncertain.

We used a difference‐in‐difference analysis to assess whether the infusion rate impacted the agreement between the methods. Although there was an overall significant difference in agreement between the fast and slow infusion groups when comparing the calibrated and reference methods, the magnitude of this difference in agreement was very small compared to the pooled LOAs. Also, this difference could not be shown at either of the specific time points. Furthermore, no difference between infusion rates could be reproduced comparing the anthropometric and reference methods. Taken together, we believe that this finding of a small effect of the infusion rate on agreement between the calibrated and reference methods represents a type I error.

### 4.1 Strengths and Limitations

Strengths of the study include the large size of the cohort and that all data were collected within the framework of a RCT with a low risk of bias. Moreover, the participants were exposed to a large volume of fluid therapy at two infusion rates covering a clinically relevant range, which supports the generalizability of the results.

Limitations include the fact that the precision of the reference method in our hands and in the current clinical context was not determined. Also, the evaluation was limited to 180 min in the present study and any use of the calibrated and the anthropometric methods beyond this time frame needs to be confirmed in future studies. Also, use of vasoactive medications in the present cohort was small suggesting a relatively mild post‐surgical SIRS. Thus, we cannot be sure that the results can be extrapolated to less homogeneous cohorts of critically ill ICU patients with more profound hemodynamic disturbances.

## 5 Conclusion

In conclusion, anthropometric data perform poorly in predicting postsurgical plasma volumes. Changes in hematocrit cannot be used to assess changes in plasma volume during and after fluid therapy in a postsurgical setting because of the imprecision of the method.

## Author Contributions

**David Grubb:** conceptualization, data analysis, drafted the original manuscript. **Svajunas Statkevicius:** data collection, revised the manuscript. **Johan Bonnevier:** data collection, revised the manuscript. **Björn Bark:** data collection, revised the manuscript. **Peter Bentzer:** conceptualization, data collection and analysis, drafted the original manuscript. All authors read and approved the final manuscript.

## Funding

Funds for this study were provided by the Swedish Government (ALF no. 86626) and the Anna and Edwin Berger Foundation.

## Conflicts of Interest

The authors declare no conflicts of interest.

## Supporting information


**Figure S1a:** Standard deviation (SD) of the plasma volumes of each measurement pair plotted against the mean of the plasma volumes between the calibrated and reference methods. Plasma volumes are normalized to predicted body weights. *N* = 128.

**Figure S1b:** Standard deviation (SD) of the plasma volumes of each measurement pair plotted against the mean of the plasma volumes between the anthropometric and reference methods. Plasma volumes are normalized to predicted body weights. *N* = 128.

**Table S1:** Difference in difference analysis.

## Data Availability

The data that support the findings of this study are available from the corresponding author upon reasonable request.

## References

[1] B. Brandstrup , H. Tönnesen , R. Beier‐Holgersen , et al., “Effects of Intravenous Fluid Restriction on Postoperative Complications: Comparison of Two Perioperative Fluid Regimens,” Annals of Surgery 238 (2003): 641–648. 14578723 10.1097/01.sla.0000094387.50865.23 PMC1356139

[2] J. Boyd , J. Forbes , T. Nakada , et al., “Fluid Resuscitation in Septic Shock: A Positive Fluid Balance and Elevated Central Venous Pressure Are Associated With Increased Mortality,” Critical Care Medicine 39 (2011): 25–265. 10.1097/CCM.0b013e3181feeb15 20975548

[3] H. Wiedemann , A. Wheeler , G. Bernard , et al., “Comparison of Two Fluid‐Management Strategies in Acute Lung Injury,” New England Journal of Medicine 354 (2006): 2564–2575. 16714767 10.1056/NEJMoa062200

[4] F. Stéphan , A. Flahault , N. Dieudonné , J. Hollande , F. Paillard , and F. Bonnet , “Clinical Evaluation of Circulating Blood Volume in Critically Ill Patients‐Contribution of a Clinical Scoring System,” British Journal of Anaesthesia 86 (2001): 754–762. 11573580 10.1093/bja/86.6.754

[5] P. Bonfils , M. Damgaard , K. Stokholm , et al., “ ^99m^Tc‐Albumin Can Replace 125I‐Albumin to Determine Plasma Volume Repeatedly,” Scandinavian Journal of Clinical and Laboratory Investigation 72 (2012): 447–451. 22646079 10.3109/00365513.2012.688856

[6] M. Margarson and N. Soni , “Plasma Volume Measurement in Septic Patients Using an Albumin Dilution Technique: Comparison With the Standard Radio‐Labelled Albumin Method,” Intensive Care Medicine 31 (2005): 289–295. 15526187 10.1007/s00134-004-2481-4

[7] T. Damén , B. Reinsfelt , B. Redfors , and A. Nygren , “Pressure‐Dependent Changes in Haematocrit and Plasma Volume During Anaesthesia, a Randomised Clinical Trial,” Acta Anaesthesiologica Scandinavica 60 (2016): 560–568. 26792419 10.1111/aas.12687

[8] D. Gillett and D. Halmagyi , “Blood Volume in Reversible and Irreversible Posthemorrhagic Shock in Sheep,” Journal of Surgical Research 6 (1966): 259–261. 5937902 10.1016/s0022-4804(66)80034-3

[9] J. Lundvall and P. Lindgren , “F‐Cell Shift and Protein Loss Strongly Affect Validity of PV Reductions Indicated by Hb/Hct and Plasma Proteins,” Journal of Applied Physiology 84 (1998): 822–829. 9480939 10.1152/jappl.1998.84.3.822

[10] S. Statkevicius , J. Bonnevier , B. Bark , et al., “The Importance of Albumin Infusion Rate for Plasma Volume Expansion Following Major Abdominal Surgery ‐ AIR: Study Protocol for a Randomised Controlled Trial,” Trials 17 (2016): 578. 27923389 10.1186/s13063-016-1714-5 PMC5142270

[11] S. Statkevicius , J. Bonnevier , J. Fisher , et al., “Albumin Infusion Rate and Plasma Volume Expansion: A Randomized Clinical Trial in Postoperative Patients After Major Surgery,” Critical Care 23 (2019): 191. 31138247 10.1186/s13054-019-2477-7 PMC6537197

[12] R. Brower , M. Matthay , A. Morris , and et al , “Ventilation With Lower Tidal Volumes as Compared With Traditional Tidal Volumes for Acute Lung Injury and the Acute Respiratory Distress Syndrome,” New England Journal of Medicine 32 (2000): 1301–1308. 10.1056/NEJM200005043421801 10793162

[13] R. Chaudhary , A. Dubey , and A. Sonker , “Techniques Used for the Screening of Hemoglobin Levels in Blood Donors: Current Insights and Future Directions,” Journal of Blood Medicine 8 (2017): 75–88. 28740442 10.2147/JBM.S103788 PMC5503668

[14] Y. Zhu and R. Hahn , “The Kinetics of Ringer's Solution in Young and Elderly Patients During Induction of General Anesthesia With Propofol and Epidural Anesthesia With Ropivacaine,” Acta Anaesthesiologica Scandinavica 51 (2007): 880–887. 17635395 10.1111/j.1399-6576.2007.01351.x

[15] S. Nadler , J. Hidalgo , and T. Bloch , “Prediction of Blood Volume in Normal Human Adults,” Surgery 51 (1962): 224–232. 21936146

[16] J. Bland and D. Altman , “Agreement Between Methods of Measurement With Multiple Observations Per Individual,” Journal of Biopharmaceutical Statistics 17 (2007): 571–582. 17613642 10.1080/10543400701329422

[17] D. Gillett and D. Halmagyi , “Accuracy of Single‐Label Blood Volume Measurement Before and After Corrected Blood Loss in Sheep and Dogs,” Journal of Applied Physiology (1985) 28 (1970): 213–215. 10.1152/jappl.1970.28.2.213 5413308

[18] T. Damén , S. Saadati , E. Forssell‐Aronsson , et al., “Effects of Different Mean Arterial Pressure Targets on Plasma Volume, ANP and Glycocalyx‐A Randomized Trial,” Acta Anaesthesiologica Scandinavica 25 (2021): 220–227. 10.1111/aas.13710 PMC7820977 32965691

[19] A. Nygren , B. Redfors , A. Thorén , and S. E. Ricksten , “Norepinephrine Causes a Pressure‐Dependent Plasma Volume Decrease in Clinical Vasodilatory Shock,” Acta Anaesthesiologica Scandinavica 54 (2010): 814–820. 20455879 10.1111/j.1399-6576.2010.02244.x

[20] B. Veering and M. Cousins , “Cardiovascular and Pulmonary Effects of Epidural Anaesthesia,” Anaesthesia and Intensive Care 28 (2000): 620–635. 11153287 10.1177/0310057X0002800603

[21] J. Bland and D. Altman , “Statistical Methods for Assessing Agreement Between Two Methods of Clinical Measurement,” Lancet (London, England) 8476 (1986): 307–310. 2868172

