# Supplementary figures and images for: Agreement Between Methods Assessing Changes in Plasma Volume During Fluid Therapy—A Post Hoc Analysis of a Randomized Trial
Source: Acta Anaesthesiol Scand. 2026 Jun 4;70(6):e70271. doi: 10.1111/aas.70271 (PMC13238360; doi:10.1111/aas.70271)

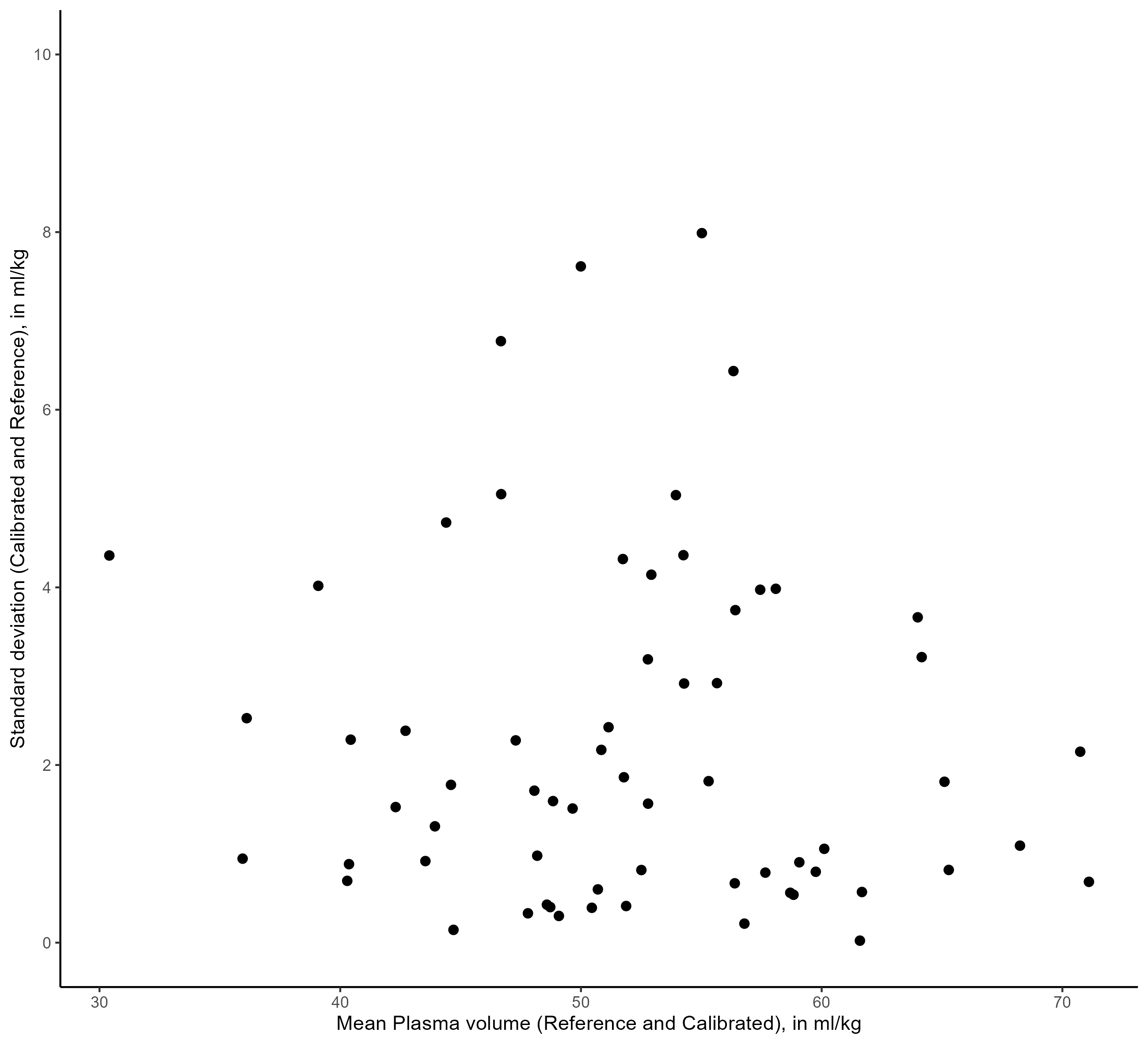

Supplement: Supplementary file 1 — Figure S1a: Standard deviation (SD) of the plasma volumes of each measurement pair plotted against the mean of the plasma volumes between the calibrated and reference methods. Plasma volumes are normalized to predicted body weights. N = 128. [file AAS-70-0-s002.gif]

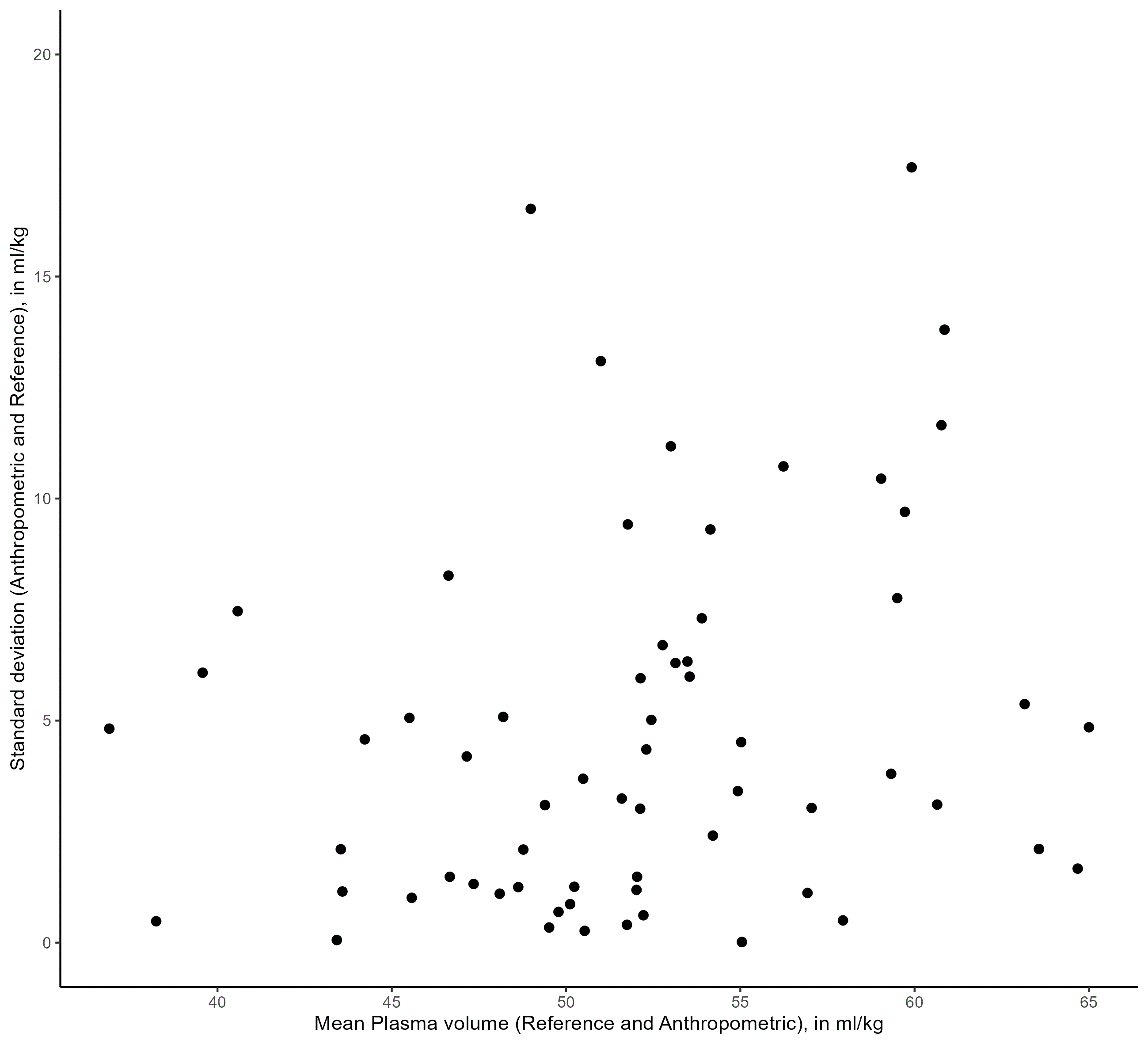

Supplement: Supplementary file 2 — Figure S1b: Standard deviation (SD) of the plasma volumes of each measurement pair plotted against the mean of the plasma volumes between the anthropometric and reference methods. Plasma volumes are normalized to predicted body weights. N = 128. [file AAS-70-0-s003.gif]
