# Supplementary material for: Agreement Between Methods Assessing Changes in Plasma Volume During Fluid Therapy—A Post Hoc Analysis of a Randomized Trial
Source: Acta Anaesthesiol Scand. 2026 Jun 4;70(6):e70271. doi: 10.1111/aas.70271 (PMC13238360; doi:10.1111/aas.70271)
Supplement: Supplementary file 3 — Table S1: Difference in difference analysis. [file AAS-70-0-s001.docx]

**Supplement table 1. Difference in Difference analysis**

|  |  | |  | Difference in Difference (95% CI) | p-value* |
| --- | --- | --- | --- | --- | --- |
| Calibrated vs Reference, ml/kg | Overall effect of infusion rate  (Slow vs Fast) | | | -1.9 (-3.5, -0.3) | 0.019 |
|  | At time = 30 | Effect of infusion rate  (Slow vs Fast) | | -2.4 (-4.5 -0.3) | 0.053 |
|  | At time = 180 | Effect of infusion rate  (Slow vs Fast | | -1.4 (-3.6, 0.7) | 0.363 |
| Anthropometric vs Reference, ml/kg | Overall effect of infusion rate  (Slow vs Fast) | | | -1.2 (-5.4; 3.1) | 0.591 |
|  | At time = 30 | Effect of infusion rate  (Slow vs Fast) | | -1.8 (-6.3, 2.7) | 0.870 |
|  | At time = 180 | Effect of infusion rate  (Slow vs Fast) | | -0.6 (-5.0, 3.9) | 1.000 |

*Bonferroni adjusted
